# *In vivo* Antidiabetic and Antioxidant Potential of *Stephania hernandifolia* in Streptozotocin-Induced-Diabetic Rats

**DOI:** 10.4103/0975-1483.66803

**Published:** 2010

**Authors:** U Sharma, RK Sahu, A Roy, DK Golwala

**Affiliations:** *Department of Pharmacognosy, Institute of Pharmacy, Jalpaiguri, West Bengal, India*; 1 *Oriental College of Pharmacy, Bhopal, Madhya Pradesh, India*; 2 *Department of Pharmacology, Columbia College of Pharmacy, Mandhar, Raipur, Chhattisgarh, India*; 3 *C.U. Shah College of Pharmacy and Research, Surendranagar-Ahmedabad Highway, Wadhwan, Gujarat, India*

**Keywords:** Antidiabetic, antioxidant, glibenclamide, lipid profile, *Stephania hernandifolia*, Streptozotocin

## Abstract

*Stephania hernandifolia* (Menispermaceae) is a medicinal plant, used by herbalists for treating various diseases, one of which is diabetes mellitus, in Darjeeling. However, its antidiabetic activity has not been scientifically investigated so far. The aim of this study, therefore, is to investigate the antidiabetic and antioxidant potential of the powdered corm of *Stephania hernandifolia*. This was tested in normal and Streptozotocin (STZ)-induced diabetic rats, using oral administration of ethanol and an aqueous extract (400 mg/kg body weight) of *Stephania hernandifolia* corm. After the oral administration of water and ethanol extracts at doses of 400 mg/kg body weight, blood glucose levels were monitored at specific intervals and it was found that they were significant lowered. Glibenclamide was used as a standard drug at a dose of 0.25 mg/kg. The experimental data revealed that both extracts has significant antihyperglycemic and antioxidant activity in Streptozotocin-induced rats compared to the standard drug. The antioxidant activity *in vitro* was measured by means of the 1, 1-diphenyl-2-picrylhydrazyl (DPPH) and Superoxide-free radical scavenging assay. Ascorbic acid, a natural antioxidant, was used as a control. The extracts of ethanol and aqueous were strongly scavenged DPPH radicals, with IC_50_ being 265.33 and 217.90 µg/ml, respectively. Although the extracts of ethanol and aqueous were moderately scavenged, the superoxide radical were with IC_50_ values of 526.87 and 440.89 µg/ml. The study revealed that the ethanolic extract exhibited more significant antidiabetic and antioxidant activity then the aqueous extract.

## INTRODUCTION

The use of herbal medicines for the treatment of diabetes mellitus has gained importance throughout the world. The World Health Organization has also recommended and encouraged this practice, especially in countries where access to the conventional treatment of diabetes is not adequate. There is an increased demand for using natural products with antidiabetic activity, on account of the side effects associated with the use of insulin and oral hypoglycemic agents.[1] The available literature shows that there are more than 400 plant species showing hypoglycemic activity.[2]

*Stephania hernandifolia* (Willd.) Walp. (Family- Menispermaceae) is a slender twining shrub found in hilly areas, above 900 meters (approximately) from the sea level. The plant is useful in the treatment of fever, diarrhea, diabetes, urinary disease, dyspepsia, depilatory.[3,4] The main objectives of this study are to assess the antidiabetic and antioxidant potential of ethanol and aqueous extracts of the powdered corm of *Stephania hernandifolia* in control of blood glucose levels and effectiveness on various biochemical parameters, namely, total cholesterol, triglycerides (TGL), high density lipoprotein (HDL), low density lipoprotein, (LDL), and very low density lipoprotein, (VLDL), with antioxidant studies.

## MATERIALS AND METHODS

### Plant material

The plant *Stephania hernandifolia* (Willd.) Walp. has been collected from Darjeeling district of West Bengal, India, during summer. The species for the proposed study was identified as *Stephania hernandifolia* (Willd.) Walp. by Dr. P. Jayaraman, Botanist, Plant Anatomy Research Centre (PARC), Chennai.

### Preparation of *Stephania hernandifolia* corm ethanolic and aqueous extracts

The powder of the corm (300 g) of *Stephania hernandifolia* (Willd.) Walp., was packed well in the Soxhlet apparatus and extracted with ethanol and distilled water separately, until the completion of the extraction. The extract was filtered while hot, and the resultant extract was distilled in vacuum under reduced pressure in order to remove the solvent completely, and later dried in a desiccator.[5]

### Animals

Male Wistar albino rats having a weight of 170 – 220 g were kept in quarantine for 10 days under standard husbandry conditions (27.3°, Relative humidity 65 ± 10%) for 12 hours in dark and light cycle, respectively, and were given standard food and water *ad libitum*. The study was permitted by the Institution Animal Ethical Committee with Reg. No. CPCSEA/265.

### Acute toxicity testing

Acute toxicity testing was performed for both ethanol and aqueous extracts following the Organization for Economic Cooperation and Development (OECD) guidelines-420, fixed dose procedure, where a fixed dose level of extracts starting from 50, 100, 200, 500, 1000, increasing up to 2000 mg/kg body weight was given, and signs and symptoms of toxicity were observed for the next 48 hours. No toxicity or death was observed in the experimental rats.[6,7]

### Oral glucose tolerance test (OGTT)

The oral glucose tolerance test[8] was performed in overnight fasted (18 hours) normal rats. The rats were divided into three groups (*n* = 6) and were administered drinking water, *Stephania hernandifolia* ethanol extract (400 mg/kg), and aqueous extracts (400 mg/kg), respectively. Glucose (2 g/kg) was fed 30 minutes prior to the administration of the extracts. Blood was withdrawn from the retro-orbital sinus after 30, 60, and 120 minutes of extract administration, and the plasma obtained after centrifugation at 3000 rpm was estimated for fasting plasma glucose levels using a glucose oxidase–peroxidase glucose estimation kit.

### Induction of non-insulin dependent diabetes mellitus (NIDDM)

Non-insulin dependent diabetes mellitus was induced[9,10] in overnight fasted adult Wistar strain albino male rats weighing 170 – 220 g by a single intraperitoneal injection of 60 mg/kg Streptozotocin, 15 minutes after i.p. administration of 120 mg/kg of nicotinamide. Streptozotocin (STZ) was dissolved in a citrate buffer (pH 4.5) and nicotinamide was dissolved in normal saline. Hyperglycemia was confirmed by the elevated glucose levels in plasma, determined at 72 hours and then on day 7, after injection. The threshold value of fasting plasma glucose to diagnose diabetes was taken as > 126 mg/dl. Only those rats that were found to have permanent NIDDM were used for the study.

### Experimental design

The animals were segregated into five groups of six rats each. The extract was administered for 12 days. Group I served as normal control rats, administered drinking water daily for 12 days; Group II had diabetic control rats, administered drinking water daily for 12 days; Group III diabetic rats were administered ethanol extract (400 mg/kg); Group IV diabetic rats were administered aqueous extract (400 mg/kg); and Group V diabetic rats were administered standard drug glibenclamide (0.25 mg/kg) for 12 days.

The fasting glucose levels were determined on days 1, 5, and 12 of extract administration. During the experimental period, the rats were weighed daily and the mean change in body weight was calculated.

### Estimation of biochemical parameters

The biochemical parameters were determined on day 12 after the animals were sacrificed by cervical dislocation. Total cholesterol, triglycerides (TGL), high-density lipoprotein (HDL), low-density lipoprotein (LDL), and very low-density lipoprotein (VLDL), were determined by the glucose oxidase method, using an auto-analyzer.[11,12]

### Determination of hydrogen-donating activity

The hydrogen donating activity was quantified in the presence of a stable DPPH radical on the basis of the Blois method. Briefly, to a methanolic solution of DPPH (100 m M, 2.95 ml), 0.05 ml of both extracts dissolved in methanol were added at different concentrations (200 – 1000 µg/ml). The reaction mixture was shaken and after 30 minutes at room temperature, the absorbance values were measured at 518 nm and converted into percentage of antioxidant activity (% AA). Ascorbic acid was used as the standard.[13,14] The degree of discoloration indicated the scavenging efficacy of the extract, which was calculated by the following equation:

$$\%\;\mathrm{AA}\;=\;100\;-\;\left\{\left[\left({\mathrm{Abs}}_{\mathrm{sample}}\;-\;{\mathrm{Abs}}_{\mathrm{blank}}\right)\;\times \;100\right]\;/\;{\mathrm{Abs}}_{\mathrm{DPPH}}\right\}$$

### Determination of the superoxide scavenging activity

Superoxide scavenging was carried out by using alkaline DMSO. Solid potassium superoxide was allowed to stand in contact with dry DMSO for at least 24 hours and the solution was filtered immediately before use. Filtrate (200 ml) was added to 2.8 ml of an aqueous solution containing nitroblue tetrazolium (56 m M), EDTA (10 mM), and potassium phosphate buffer (10 mM, pH 7.4). Sample extract (1 ml) in various concentrations (200 – 1000 µg/ml) in water was added and the absorbance was recorded at 560 nm against a control in which pure DMSO was added instead of alkaline DMSO.[15,16]

### Statistical analysis

The results are expressed as mean ± SEM of six independent experiments. Statistical significance between the groups was evaluated by one-way analysis of variance (ANOVA) followed by Dunet’s test. A P < 0.05 value was considered as statistically significant. For antioxidant activity, values representing the concentrations of investigated extracts that cause 50% of neutralization / inhibition (IC_50_) were determined by linear regression analysis.

## RESULTS

The effects of ethanol and aqueous extracts of *Stephania hernandifolia* on the plasma glucose level are shown in Table 1. In rats treated with both the extracts, there was a significant reduction in plasma glucose level, while in normal control rats the plasma glucose level increased. Induction of diabetes in experimental rats was confirmed by the presence of a high fasting plasma glucose level. The effect of both extracts of *Stephania hernandifolia*, on fasting plasma glucose level of normal and Streptozotocin-induced rats are shown in Table 2.

**Table 1:** Effect of ethanol and aqueous extracts of *Stephania hernandifolia* on oral glucose tolerance test

Group	Plasma glucose concentration (mg/dl)		
	30 min	60 min	90 min
Normal control	72.66 ± 1.75	97.17 ± 2.28	95.2 ± 1.94
Normal + EESH (400 mg/kg)	64.68 ± 2.13*	82.52 ± 2.19*	70.1 ± 1.18*
Normal + AESH (400 mg/kg)	69.12 ± 1.37	87.92 ± 1.78*	72.55 ± 1.14*

Ethanol extract of *Stephania hernandifolia* (EESH), Aqueous extract of *Stephania hernandifolia* (AESH), Values are expressed as mean ± SEM (Number of animals, n = 6);

* Significantly different from the normal control at *P* < 0.05

**Table 2:** Effect of ethanol and aqueous extracts of *Stephania hernandifolia* on fasting plasma glucose level in rats

Group	Fasting plasma glucose concentration (mg/dl)		
	Day 1	Day 5	Day 12
Normal control	75.91 ± 1.65	77.01 ± 1.88	75.61 ± 1.41
Diabetic control (Streptozotocin)	189.17 ± 2.35*	192.3 ± 1.41*	202.4 ± 2.40*
Diabetic + EESH (400 mg/kg)	188.53 ± 1.99*	108.23 ± 2.78*	90.51 ± 2.97*
Diabetic + AESH (400 mg/kg)	191.38 ± 2.16*	122.6 ± 2.91*	99.47 ± 3.01
Diabetic + Standard Glibenclamide (0.25 mg/kg)	192.11 ± 2.68*	98.17 ± 1.73*	80.9 ± 1.84*

Ethanol extract of *Stephania hernandifolia* (EESH), Aqueous extract of *Stephania hernandifolia* (AESH), Values are expressed as mean ± SEM (Number of animals, n = 6);

* Significantly different from the normal control at *P* < 0.05

The difference between the experimental and control rats in lowering the fasting plasma glucose levels was statistically significant (*P* < 0.05) in diabetic rats. From Table 2 it was seen that the decrease in glucose level with both extracts was less when compared to that with the standard drug. Significant differences were also observed in the level of total cholesterol, HDL, triglycerides, VLDL, and LDL in diabetic rats [Table 3]. During the study, the body weight of diabetic animals that were treated with extracts and the standard drug, throughout the twelve days was also followed and no significant change was observed [Table 4].

**Table 3:** Determination of biochemical parameters after treatment with ethanol and aqueous extracts of *Stephania hernandifolia* and Glibenclamide

Group	Total cholesterol (mg/dl)	HDL (mg/dl)	Triglycerides (mg/dl)	VLDL (mg/dl)	LDL (mg/dl)
Normal control	80.0 ± 3.01	49.73 2.55	81.47 ± 2.61	29.99 ± 2.71	39.11 ± 3.73
Diabetic control (Streptozotocin)	149.3±4.04*	46.72 1.99	177.8 ± 2.91*	64.08 3.74*	155.2±4.45*
Diabetic + EESH (400 mg/kg)	69.44 ± 2.84	43.63 2.81	98.5 ± 3.38*	23.72 ± 2.40	55.4 ± 3.96*
Diabetic + AESH (400 mg/kg)	74.85 ± 4.32	44.61 3.37	103.61 4.52*	25.02 ± 3.45	82.84±2.81*
Diabetic + Standard Glibenclamide (0.25 mg/kg)	59.23±3.12*	43.46 3.79	88.16 ± 3.52*	28.62 ± 3.23	32.96 3.66*

Ethanol extract of *Stephania hernandifolia* (EESH), Aqueous extract of *Stephania hernandifolia* (AESH), Values are expressed as mean ± SEM (Number of animals, n = 6);

* Significantly different from the normal control at *P* < 0.05

**Table 4:** Effect of ethanol and aqueous extracts of *Stephania hernandifolia* on changes in body weight in rats

Group	Day 1	Day 5	Day 12
Normal control	176.72±2.23	182.28±1.40	173.54±3.71
Diabetic control (Streptozotocin)	190.47±3.71	185.81±2.87	168.16±2.47
Diabetic + EESH (400 mg/kg)	188.29±2.56	185.46±3.04	179.35±1.12
Diabetic + AESH (400 mg/kg)	184.31±1.93	186.73±4.11	177.82±3.54
Diabetic + Standard Glibenclamide (0.25 mg/kg)	191.5 ± 4.12	188.35±2.74	180.81±2.87

Ethanol extract of *Stephania hernandifolia* (EESH), Aqueous extract of *Stephania hernandifolia* (AESH), Values are expressed as mean ± SEM (Number of animals, n = 6)

**Figure 1:**
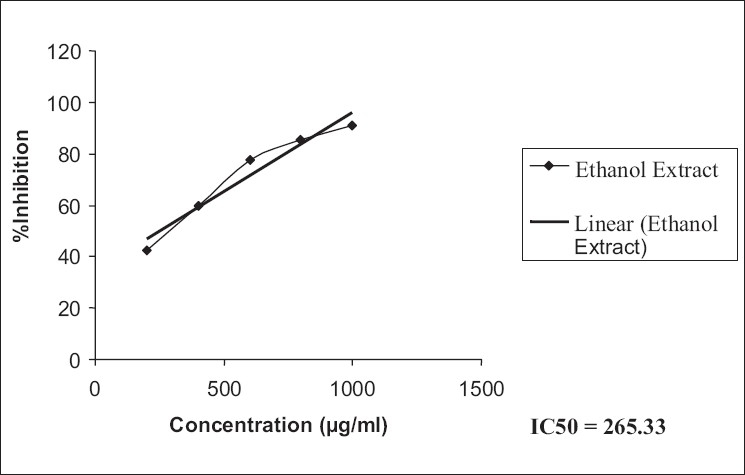
IC_50_ values of ethanol extract of free radical scavenging capacity, from the data, were calculated by regression analysis

**Figure 2:**
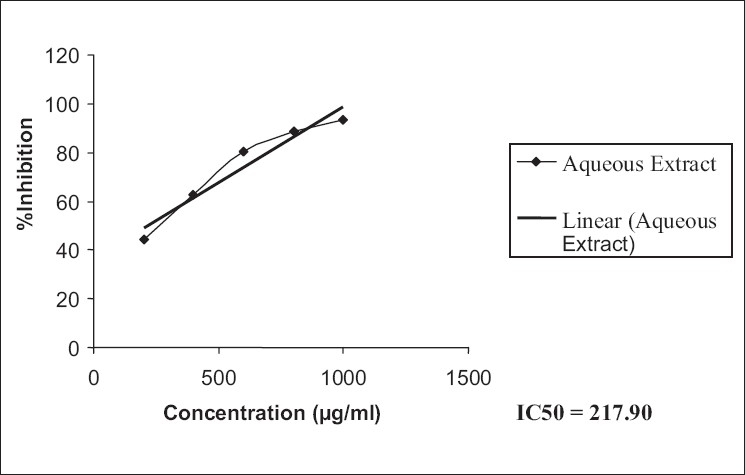
IC_50_ values of aqueous extract of free radical scavenging capacity, from the data, were calculated by regression analysis

**Figure 3:**
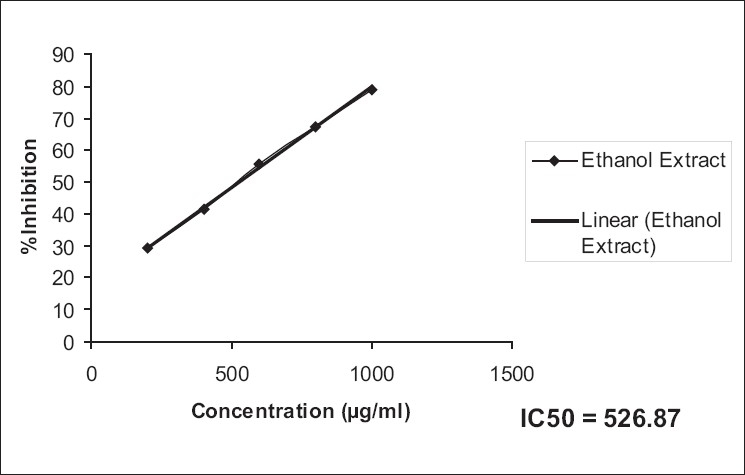
IC_50_ values of ethanol extract of superoxide scavenging capacity, from the data, were calculated by regression analysis

**Figure 4:**
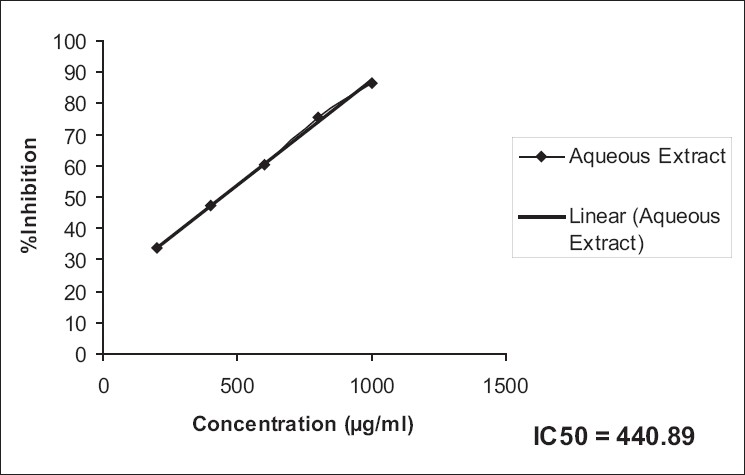
IC_50_ values of aqueous extract of superoxide scavenging capacity, from the data, were calculated by regression analysis

The ethanol extract possessed a higher antioxidant activity compared to that of the aqueous extract in DPPH [IC_50_: EESH versus AESH; 265.33 versus 217.90, Table 5, Figures 1, 2] as also superoxide scavenging capacity [IC_50_: EESH versus AESH; 526.87 versus 440.89, Table 6, Figures 3, 4].

**Table 5:** Free radical scavenging capacity of ethanol and aqueous extracts of *Stephania hernandifolia*

Concentration (µg/ml)	DPPH Scavenging %		
	Ethanol Extract	Aqueous Extract	Ascorbic Acid
200	42.65 ± 0.58	44.11 ± 1.68	94.35 ± 1.25
400	59.58 ± 1.14	62.41 ± 1.75	-
600	77.45 ± 1.85	80.52 ± 1.96	-
800	85.32 ± 2.45	88.56 ± 1.37	-
1000	91.15 ± 1.32	93.12 ± 0.85	-
IC_50_	265.33	217.90	-

Values are means ± SEM of six determinations

**Table 6:** Super oxide scavenging capacity of ethanol and aqueous extracts of *Stephania hernandifolia*

Concentration (µg/ml)	Superoxide Scavenging %		
	Ethanol Extract	Aqueous Extract	Ascorbic Acid
200	29.23 ± 0.47	33.75 ± 1.25	89.41 ± 1.52
400	41.39 ± 1.08	47.23 ± 2.36	-
600	55.85 ± 0.96	60.25 ± 2.41	-
800	67.45 ± 1.48	75.45 ± 2.16	-
1000	79.12 ± 1.56	86.32 ± 2.04	-
IC_50_	526.87	440.89	-

Values are means ± SEM of six determinations

## DISCUSSION

The fundamental mechanism underlying hyperglycemia in diabetes mellitus involves over-production and decreased utilization of glucose by the tissues. In our study, the difference observed between the initial and final fasting plasma glucose levels of different groups under investigation, revealed a significant elevation in blood glucose in the diabetic control group as compared to normal animals, at the end of the twelve-day experimental period. When ethanol and aqueous extracts of *Stephania hernandifolia* were administered to glucose-loaded normal rats, fasted for 18 hours, a decrease in plasma glucose level was observed after 30 minutes. Both the extracts reduced plasma glucose level to normal at 90 minutes [Table 1]. During the study it was found that both extracts significantly controlled the blood glucose level in Streptozotocin-induced diabetic rats. The ethanol and aqueous extracts induced a significant reduction in blood glucose level in STZ-induced-diabetic rats as compared to the diabetic control group [Table 2]. However, the ethanol extract showed more significant antidiabetic activity as compared to the aqueous extract. The possible mechanism by which *Stephania hernandifolia* brought about its hypoglycemic action in diabetic rats might be by potentiating the insulin effect of plasma, by increasing either the pancreatic secretion of insulin from the existing beta cells or by its release from the bound form. A marked increase in serum triglycerides and cholesterol was observed in untreated diabetic rats. Under normal circumstances insulin activated enzyme lipoprotein lipase and hydrolyses triglycerides. Insulin deficiency resulted in failure to activate the enzymes thereby causing hypertriglyceridemia. A significant control of the levels of serum lipids in diabetic rats treated with both the extracts could be directly attributed to improvements in insulin levels upon *Stephania hernandifolia* therapy. Elevation of plasma lipid concentration in diabetes is well-documented. In insulin-deficient diabetics, the plasma-free fatty acid concentration is elevated as a result of increased free fatty acid outflow from the fat depots, where the balance of the free fatty acid esterification–triglyceride lipolysis cycle is displaced in favor of lipolysis. Induction of diabetes with STZ is associated with characteristic loss of body weight, which is due to increased muscle wasting in diabetes.[17] Diabetic rats treated with the extracts showed an increase in body weight when compared with the diabetic control, which may be due to its protective effect in controlling muscle wasting, that is, reversal of gluconeogenesis.

Abnormalities in lipoproteins are very common in both NIDDM and IDDM. Although lipoprotein alterations appear to be an intrinsic part of these disorders, such alterations are also induced by diabetes-associated complications such as obesity and renal disease.[18] The total cholesterol, triglyceride levels, VLDL, and LDL were observed to be elevated in diabetics, but reduced by both extracts as well as glibenclamide, showing their beneficial effects. In the present study, HDL levels remained unchanged in diabetics compared to the other groups [Table 3]. These results suggest that the beneficial effects of the natural extract in improving the imbalance in lipoprotein metabolism are also comparable to those of glibenclamide.

In recent times, there has been a considerable debate regarding the extent to which increased oxidative stress contributes toward the development of diabetic complications. The role of antioxidant compounds in both the protection and therapy of diabetes mellitus was also emphasized in previous scientific researches. Hyperglycemia results in the generation of free radicals that can exhaust antioxidant defenses thus leading to the disruption of cellular functions, oxidative damage to membranes, and enhanced susceptibility to lipid peroxidation. Flavonoids are one of the most numerous and widespread groups of phenolics in plants. Some of them, due to their phenolic structure, are known to be involved in the healing process of free radical-mediated diseases, including diabetes.[19] In our study, the ethanol and aqueous extracts were found to have a strong antioxidant activity. This may be due to the presence of phenols and flavonoids, which may have a major role in reducing oxidative stress associated with diabetes. Thus the significant antidiabetic activity of ethanol and aqueous extracts of *Stephania hernandifolia* in our study, may be attributed to the presence of flavonoids in the plant.

The present study has indicated the fact that the plant *Stephania hernandifolia* (Willd.) Walp., has antidiabetic constituents and production of a safe antidiabetic drug is very much possible from the corm.
